# Gut Microbial Signatures Associated with Peanut Allergy in a BALB/c Mouse Model

**DOI:** 10.3390/foods11101395

**Published:** 2022-05-12

**Authors:** Shimin Gu, Qiang Xie, Chen Chen, Chenglong Liu, Wentong Xue

**Affiliations:** College of Food Science & Nutritional Engineering, China Agricultural University, Beijing 100083, China; g315035195@163.com (S.G.); xq294134@126.com (Q.X.); 15515658204@163.com (C.C.); chenglong611@163.com (C.L.)

**Keywords:** peanut, allergy, gut microbiota, 16S rRNA sequencing, predicted functions

## Abstract

Multiple studies have uncovered the pivotal role of gut microbiota in the development of food allergy. However, the effects of gut microbiota on peanut allergy are still unclear. Here, we characterized the gut microbiota composition of peanut-allergic mice by 16S rRNA sequencing and analyzed the correlation between allergic indicators and gut microbiota composition. Outcomes showed that the gut microbiota composition was reshaped in peanut-allergic mice, with Acidobacteriota, Lachnospiraceae, Rikenellaceae, *Alistipes*, *Lachnospiraceae_NK4A136_group* significantly down-regulated and Muribaculaceae up-regulated. All of them were significantly correlated with the serum peanut-specific antibodies. These results suggested that these six bacterial OTUs might be the gut microbial signatures associated with peanut allergy.

## 1. Introduction

Food allergy (FA) is a serious public health issue, with growing prevalence over the decades [1]. According to the National Institute of Allergy and Infectious Diseases, peanuts, nuts, seafood, milk, and eggs are regarded as the most common allergens responsible for most FA incidents [2]. About 0.6% of people in the United States suffer from peanut allergy (PA) [2]. PA is usually lifelong and induces various anaphylaxis including urticaria, angioedema, asthma, hypotension, and cardiac arrest, some of which are life-threatening [3,4]. However, the immune mechanisms of PA are still unclear.

Multiple studies have shown a critical role of gut microbiota in FA development [5,6]. People with FA demonstrated distinct gut microbiota compared to the healthy controls [7]. The imbalance of Bacteroidetes and Firmicutes might contribute to FA [8]. Mice devoid of commensal microbiota had increased susceptibility to FA, while the colonization of Clostridia-containing microbiota reversed the susceptibility [9]. Moreover, the gut microbiota can strengthen the tight junction between intestinal epithelial cells (IECs) by inducing the dendritic cells and macrophages to produce interleukin (IL)-22, thus improving the intestinal mucosal barrier and protecting against FA [9]. Short-chain fatty acid (SCFA) produced by some specific bacteria including *Bacteroides*, *Lactobacillus*, *Bifidobacterium*, and *Alloprevotella* can intervene in metabolism and thereby provide energy to IECs [10,11,12]. Beyond that, the gut microbiota has the potential to regulate T cell differentiation. Newborn infants showed a helper T cell (Th) 2-biased immune response for lack of diversity in microbiota [13,14]. This imbalance was subsequently restored by the colonization of 10^14^ bacteria from the introduction of oral feedings [15]. It was also reported that the mixture of Clostridia strains from human microbiota enhanced the expression of regulatory T cells and alleviated the symptoms in colitis mice [16]. These findings elucidated how gut microbiota affected host immune homeostasis. 

This study explored the association between PA and gut microbiota in a BALB/c mouse model. The gut microbiota was characterized by 16S rRNA sequencing. The correlation between allergic indicators and gut microbiota composition was also analyzed to identify the PA-associated gut microbial signatures. The objectives were to raise new evidence for the identification and classification of characteristic PA-related microbiota and provide additional perspectives for interpreting the PA-gut microbiota interaction.

## 2. Materials and Methods 

### 2.1. Materials 

Peanut protein was purchased from Shanghai Yuanye Biotechnology Co., Ltd. BALB/c mice (6 weeks old, female) were provided by Si Pei Fu Inc (Beijing, China). The Bradford protein assay kit and Bacterial Genomic DNA Extraction kit were purchased from Beijing Solarbio science & technology Co., Ltd. (Beijing, China). Rat anti-mouse (Immunoglobulin, Ig) E, IgG2a, and IgA were purchased from Abcam Co., Ltd. (Cambridge, MA, USA). IL-4 enzyme-linked immunosorbent assay (ELISA) kit was obtained from Invitrogen by Thermo Fisher Scientific (Waltham, MA, USA).

### 2.2. Preparation of Peanut Protein Extraction

The peanut protein extracts (PE) were prepared as described by Koppelman et al. [17]. Briefly, the peanut protein was extracted by mixing 25 g of peanuts with 250 mL of 20 mM Tris buffer (pH 7.2). After 2 h stirring at room temperature, the aqueous fraction was collected by centrifugation at room temperature (3000× *g* for 30 min). The aqueous fraction was centrifuged again at room temperature (10,000× *g* for 30 min) to remove residual traces of fat and insoluble particles. The peanut protein extracts contained 18 mg/mL protein, which was determined by Bradford analysis with bovine serum albumin (BSA) as a standard. The peanut protein extracts were stored at −80 °C. 

### 2.3. SDS-PAGE Analysis

PE samples containing 5 μg, 10 μg, and 15 μg protein were mixed with loading buffer and heated together at 100 °C for 5 min. Samples and markers (14–100 kDa) were loaded into the gel. After the electrophoresis was performed at 90 v for 1 h and 180 v for 3h, the protein bands were visualized by brilliant blue (R-250) for 1 h. Then, the gel was destained in washing buffer for the whole night. The protein images were taken by the gel documentation system GenoSens 1880.

### 2.4. Ethics Statement 

This study was performed in strict accordance with the recommendations of the National Guide for the Care and Use of Laboratory Animals of China. All animal procedures were approved by the Beijing Municipal Science and Technology Commission of China (NO. SYXK 2010-0036). 

### 2.5. Mice

Six-week-old specific-pathogen-free (SPF) BALB/c female mice were obtained from Si Pei Fu Inc (Beijing, China). Mice were used for our protocols after one-week acclimation to the new environment. Mice were maintained under pathogen-free conditions of temperature (23 ± 2 °C)/humidity (40–70%) and provided with free water and food. 

### 2.6. Immunization Protocols 

Mice were randomly divided into three groups: control (Ctrl) group (*n* = 6), adjuvant (Ad) group (*n* = 6), and PE-sensitized (PE) group (*n* = 6). The peanut immunization protocols were revised from a previous study [18]. Mice in the PE group were received 0.5 mg PE (described above) adsorbed to 2 mg aluminum hydroxide (alum) (Imject™ Alum, Thermo Scientific, Waltham, MA, USA) on days 1, 7, and 21 via intraperitoneal injection and challenged twice with 15 mg PE via gavage on day 28 and 2 mg PE via intraperitoneal injection on day 35. The Ad group and Ctrl group were administered with the same volume of alum and sterile phosphate buffer saline (PBS), respectively. The clinical symptoms of mice were evaluated by allergy symptom score one hour after the intraperitoneal challenge. Body temperature was monitored by rectal thermometer 30 min before and after the intraperitoneal challenge. Mice were sacrificed on day 36. Blood samples were obtained from retro-orbital venous plexus to determine the levels of PE-specific IgE (sIgE), PE-specific IgG2a (sIgG2a), and PE-specific IgA (sIgA) on day 36. The gut tissue and spleen of mice were collected for further analysis. The allergy symptom score was graded as follows [19]: 0 = no symptom; 1 = scratching around the nose and head; 2 = puffiness around the eyes and mouth; 3 = wheezing, difficulty breathing; 4 = no activity after prodding; and 5 = death. 

### 2.7. Measurement of Levels of sIgE, sIgG2a, and sIgA in Serum

Blood samples were obtained the day after the intraperitoneal challenge from the retro-orbital venous plexus. The serum was collected by centrifugation at 3000× *g* for 10 min at 4 °C. The levels of sIgE, sIgG2a, and sIgA were measured by ELISA as previously described with some modifications [20]. Briefly, plates were coated with PE (10 μg/mL) dissolved in PBS (pH 7.4) overnight at 4 °C and blocked with 1% BSA/PBS for 1 h at 37 °C. Diluted serum samples were added to the plates and incubated for 2 h at 37 °C. Then, plates were incubated for 1 h at 37 °C with HRP-conjugated anti-mouse IgE, IgG2a, and IgA (Abcam, Cambridge, MA, USA), followed by the addition of TMB (Beyotime, Shanghai, China). The reaction was stopped by H_2_SO_4_. The absorbance was determined at 450 nm. The plates were washed between each incubation.

### 2.8. Cytokine Production in Duodenum and Spleen 

The level of IL-4 in the duodenum in mice was measured as previously described with some modifications [21]. Parts of the duodenal tissue were homogenized with PBS (pH 7.4) and centrifuged at 4 °C for 10 min at 13,000 rpm. Then, the supernatant was collected and stored at −20 °C. The measurement of the level of IL-4 in the spleen in mice was described in a previous study [21]. The levels of IL-4 in the supernatants were determined by ELISA kits (Invitrogen, Thermo Fisher Scientific, Waltham, MA, USA) following the manufacturer’s recommendations.

### 2.9. Pathological Analysis of Duodenal Tissue in Mice

Parts of the duodenal tissue were stained with hematoxylin−eosin (H&E) and Toluidine blue for pathological analysis and mast cells count, respectively.

### 2.10. 16S rRNA Gene Sequencing

The microbial genome of the colonic contents was obtained by a Bacterial Genomic DNA Extraction kit (Solarbio, Beijing, China). The V3−V4 regions of the 16S rDNA were amplified with the primers by polymerase chain reaction. NovaSeq6000 was used for on-machine sequencing. Operational Taxonomic Units (OTUs) with an identity of 97% were clustered based on Uparse (Version 7.0.1001) [22]. Alpha diversity indices (α-diversity indices) were calculated by Qiime software (Version 1.9.1) [23]. Principal coordinates analysis (PCoA) was conducted by R software (Version 2.15.3) [24] based on weighted UniFrac distances. MetaStat analysis conducted by R software was used to obtain the *p* value among groups. Then, Benjamini-Hochberg false discovery rate was used to correct the *p* value. Finally, the *q* value was obtained [25]. Environmental factor correlation analysis was performed based on Spearman correlation analysis. The Tax4Fun function prediction was conducted to predict the gene functions based on the SILVA SSU Ref NR database and Kyoto Encyclopedia of Genes and Genomes (KEGG) database.

### 2.11. Statistical Analysis

All test data were shown as means ± standard deviation (SD). Data differences among the different groups were analyzed using a one-way ANOVA with Tukey’s multiple comparisons test based on GraphPad Prism v8 (San Diego, CA, USA).

## 3. Results

### 3.1. SDS-PAGE Analysis of PE

**Figure 1:**
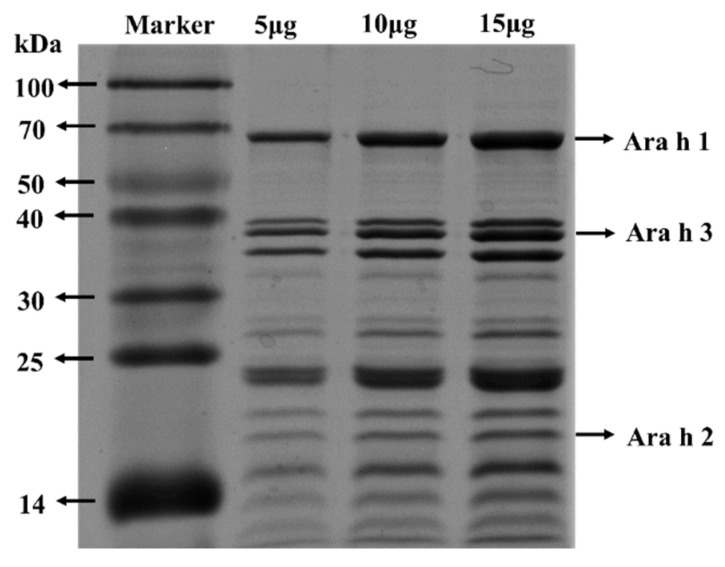
SDS−PAGE analysis of PE.

The protein components of PE were analyzed by SDS-PAGE. As illustrated in Figure 1, 15 μg, 10 μg, and 15 μg PE were loaded. Ara h 1 (63 kDa), Ara h 2 (17 kDa), and Ara h 3 (35–45 kDa) are the dominant allergens in peanuts, which can be recognized by IgE in over 50% of PA patients [26,27,28,29,30]. These three main peanut allergens could be significantly observed in PE. The contents of Ara h 1 and Ara h 3 were higher than those of Ara h 2. PE also contained other peanut allergens such as Ara h 5 (14 kDa), Ara h 6 (16 kDa), Ara h 12 (10–12 kDa), and so on [31,32].

### 3.2. PE Sensitization Caused Severe Allergic Responses in Mice

**Figure 2:**
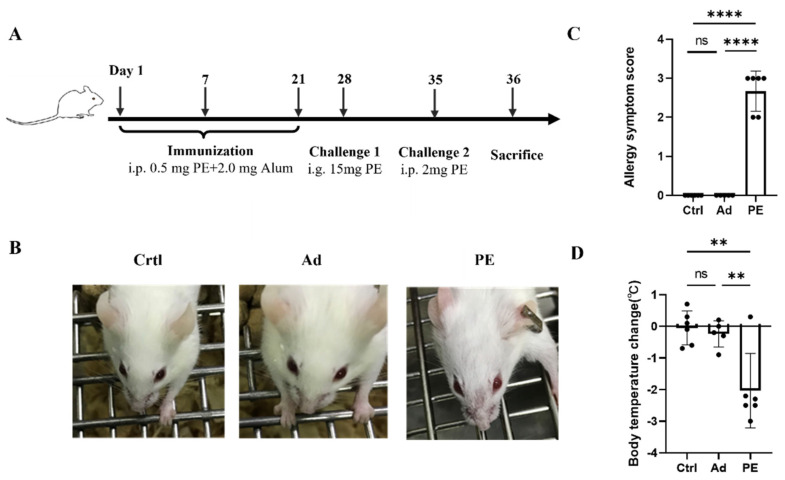
The allergic responses of mice (*n* = 6/group). (**A**) Immunization protocol for PE sensitization in BALB/c mice. (**B**) Allergic symptoms in mice. (**C**) Allergy symptom score. (**D**) Body temperature change. i.p., intraperitoneal injection; i.g., oral gavage. ns: no significance; **: *p* < 0.01; ****: *p* < 0.0001.

As shown in Figure 2B, mice sensitized with PE exhibited severe allergic symptoms including scratching and puffiness around the noses and high respiratory rates. Meanwhile, mice in the Ad and Ctrl group showed no sign of PE sensitization, which was consistent with the allergy symptom score (Figure 2C). The body temperature of mice was measured 30 min before and after the intraperitoneal challenge. A significant decrease in body temperature was observed in PE-sensitized mice compared to the mice in the Ad group (*p* < 0.01) (Figure 2D).

### 3.3. PE Sensitization Increased the Levels of Peanut-Specific Antibodies and IL-4

**Figure 3:**
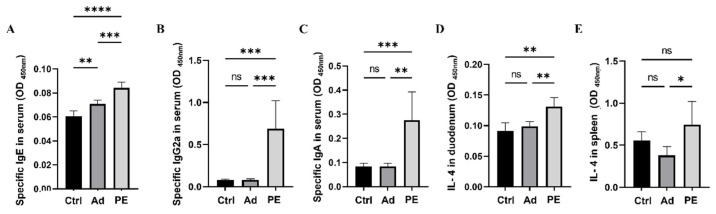
The levels of peanut-specific antibodies in serum and IL-4 in duodenum and spleen in mice (*n* = 6/group for peanut-specific antibodies, *n* = 5/group for IL-4 in duodenum and spleen). (**A**) The level of sIgE in serum. (**B**) The level of sIgG2a in serum. (**C**) The level of sIgA in serum. (**D**) The level of IL-4 in duodenum. (**E**) The level of IL-4 in spleen. ns: no significance; *: *p* < 0.05; **: *p* < 0.01; ***: *p* < 0.001; ****: *p* < 0.0001.

Allergen-specific antibodies including sIgE, sIgG2a, and sIgA are critical allergic indicators for assessing allergen sensitization [33]. They mediate allergic reactions and play a key role in recognizing different epitopes on allergens [34,35]. The levels of sIgE, sIgG2a, and sIgA in serum were measured 24 h after the intraperitoneal challenge. All of them were significantly increased in PE-sensitized mice compared to the mice in the Ad group (*p* < 0.01) (Figure 3A–C). The level of sIgE was higher in the Ad group compared to the Ctrl group, which might be because the alum could boost IgE in type I allergy [36].

To further assess the allergic reactions in mice, cytokine levels in the duodenum and spleen were studied. IL-4 is regarded as a Th2-related cytokine [37]. PE sensitization significantly enhanced IL-4 levels both in the duodenum and spleen compared to the Ad group (*p* < 0.05) (Figure 3D,E). Taken together, PE sensitization induced severe allergy symptoms, antigen-specific antibodies, and pro-inflammatory cytokines in mice.

### 3.4. PE Sensitization Impaired the Intestinal Mucosa Barrier and Promoted Mast Cell Activation in Mice

**Figure 4:**
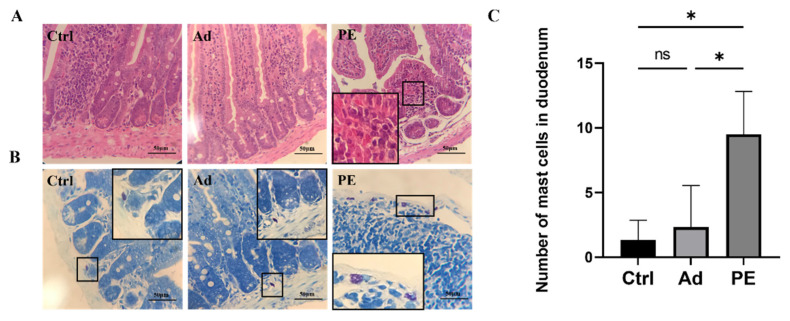
The representative photomicrographs of duodenal tissue in mice (*n* = 4/group). (**A**) H&E-stained duodenal tissue; magnifications, ×400. (**B**) Toluidine blue-stained duodenal tissue; magnifications, ×400. (**C**) The number of mast cells in the duodenum. ns: no significance; *: *p* < 0.05.

Next, we evaluated the impact of PE sensitization on the intestinal mucosa barrier function in mice. According to the duodenal tissue stained by H&E, the PE-sensitized mice presented inflammatory cell infiltration and severe tissue lesions including decreased muscular layer width and crypt depth in the duodenum (Figure 4A). The duodenal tissue was also stained by Toluidine blue (Figure 4B). As expected, the mast cells in the Ctrl and Ad groups were clearly outlined, with no degranulation observed. However, the PE group aggravated the infiltration and degranulation of mast cells. Mast cells in PE-sensitized mice were ruptured, accompanied with the appearance of granules. The number of mast cells in the PE group was significantly increased in duodenal tissue compared to the Ad group (*p* < 0.05) (Figure 4C). Altogether, PE sensitization not only impaired the small intestinal mucosa barrier but also induced mast cell degranulation.

### 3.5. PE Sensitization Changed Microbiota Diversity and Structure

**Figure 5:**
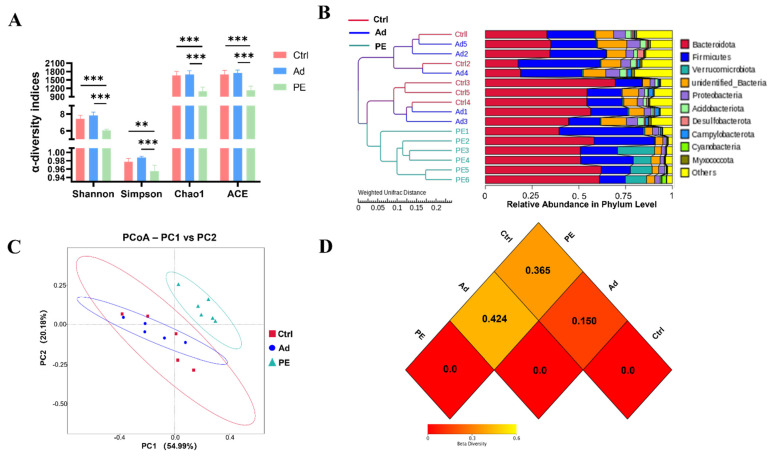
The diversity of colonic gut microbiota in mice (*n* = 6 for PE group; *n* = 5 for Ad and Ctrl groups). (**A**) α−diversity indices were assessed by Shannon, Simpson, Chao1, and ACE. β−diversity indices were assessed by the cluster tree at the phylum level (**B**), PCoA analysis (**C**), and heatmap (**D**) based on weighted UniFrac distances. Each dot represented one sample. ns: no significance; **: *p* < 0.01; ***: *p* < 0.001.

16S rRNA gene sequencing analysis was performed to assess the effect of PE sensitization on the colonic gut microbiota. Shannon, Simpson, Chao1, and ACE indices are commonly used to assess α-diversity. Shannon and Simpson indices represent the community diversity. Chao1 and ACE indices represent the community richness [38]. It was noticed that the PE group had a significant decrease in the community richness and community diversity of gut microbiota compared to the Ad group (*p* < 0.001) (Figure 5A).

β-diversity was assessed by the cluster tree, PCoA analysis, and heatmap based on weighted UniFrac distances. The cluster tree at the phylum level was divided into two main branches (Figure 5B). One was the PE group, and the other was the mixed group with the Ad and Ctrl groups. It suggested that the Ad and Ctrl groups had similar gut microbiota structures. The addition of the alum might hardly affect gut microbiota structure. Consistently, the PCoA result indicated that the PE group had a great distinction from the Ad and Ctrl groups (Figure 5C). The heatmap of β-diversity also illustrated the obvious difference between the PE and Ad groups. A smaller dissimilarity coefficient between the two groups indicates a smaller difference in species diversity. The PE and Ad groups presented the greatest difference with 0.424 (Figure 5D). Taken together, PE sensitization resulted in a reduced α-diversity and altered gut microbiota structure.

### 3.6. PE Sensitization Altered the Gut Microbiota Composition at Different Taxonomic Levels

**Figure 6:**
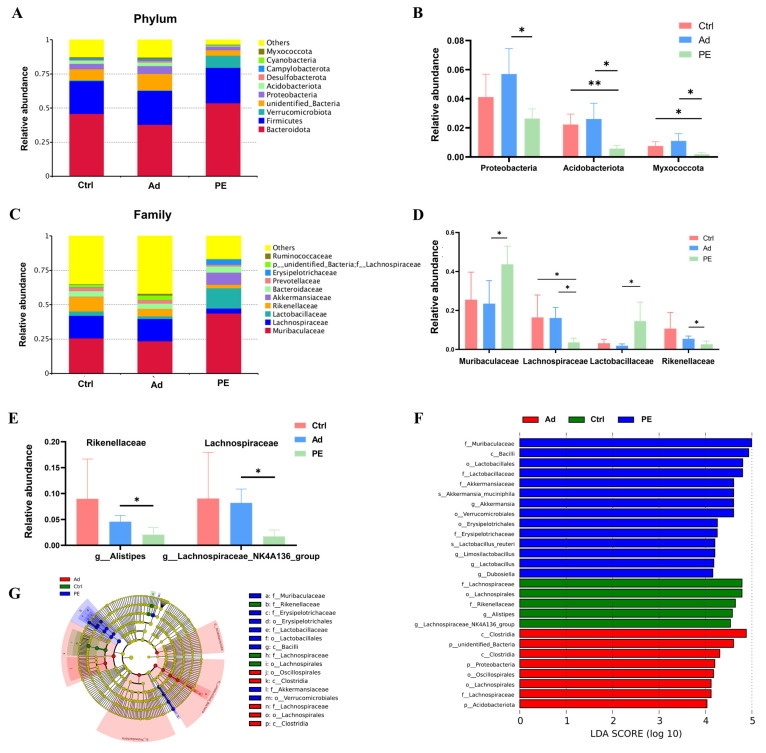
The composition of gut microbiota at different taxonomic levels in the colons in mice (*n* = 6 for PE group; *n* = 5 for Ad and Ctrl groups). (**A**) Relative abundance of gut microbiota at the phylum level. (**B**) Relative abundance of bacterial phyla with significant differences based on the *q* value. (**C**) Relative abundance of gut microbiota at the family level. (**D**) Relative abundance of bacterial families with significant differences based on the *q* value. (**E**) Relative abundance of *Alistipes* and *Lachnospiraceae_NK4A136_group*. (**F**) LDA score (LDA score threshold > 4). (**G**) Taxonomic cladogram. ns: no significance; *: *q* < 0.05; **: *q* < 0.01.

We further analyzed the relative abundance of gut microbiota at various taxonomic levels (phylum, family, and genus levels). Since there were so many species with significant differences based on a *p* value, we used a *q* value to correct the *p* value and obtain more representative species. Finally, the species with significant differences were screened based on the *q* value (Figure 6B,D,E). Overall, the dominant microbiota at the phylum level in these three groups was Bacteroidota and Firmicutes. The PE group showed increased relative abundance of Bacteroidota (*q* > 0.05) (Figure 6A). The levels of Proteobacteria, Acidobacteriota, and Myxococcota were significantly decreased in the PE group compared to the Ad group (*q* < 0.05) (Figure 6B). The LDA scores of Proteobacteria and Acidobacteriota were over 4, which indicated that the two bacteria phyla were closely associated with PA (Figure 6F). Muribaculaceae, Lachnospiraceae, Lactobacillaceae, and Rikenellaceae were the top four bacteria families in abundance among the three groups (Figure 6C). As expected, PE sensitization resulted in significant alterations in these four bacteria families. The relative abundance of Muribaculaceae and Lactobacillaceae was increased while that of Lachnospiraceae and Rikenellaceae was decreased in the PE group (*q* < 0.05) (Figure 6D). Additionally, the LDA scores of these four bacteria families were over 4 (Figure 6F). Then, we further explored the key genera in these four bacteria families. The level of *Roseburia* and *Rikenella* was significantly decreased in the PE group compared to Ctrl group while there was no significant difference between the PE group and Ad group (Figure S1). Of note, there was a significant decrease in the relative abundance of *Alistipes* belonging to Rikenellaceae and *Lachnospiraceae_NK4A136_group* belonging to Lachnospiraceae in the PE group compared to the Ad group (*q* < 0.05) (Figure 6E). 

It implied that they might have a potential association with PA. These results confirmed that PE sensitization altered the gut microbiota composition, which might contribute to developing the gut microbial signatures of PA.

### 3.7. The Correlation between Gut Microbiota and Serum Peanut-Specific Antibodies

**Figure 7:**
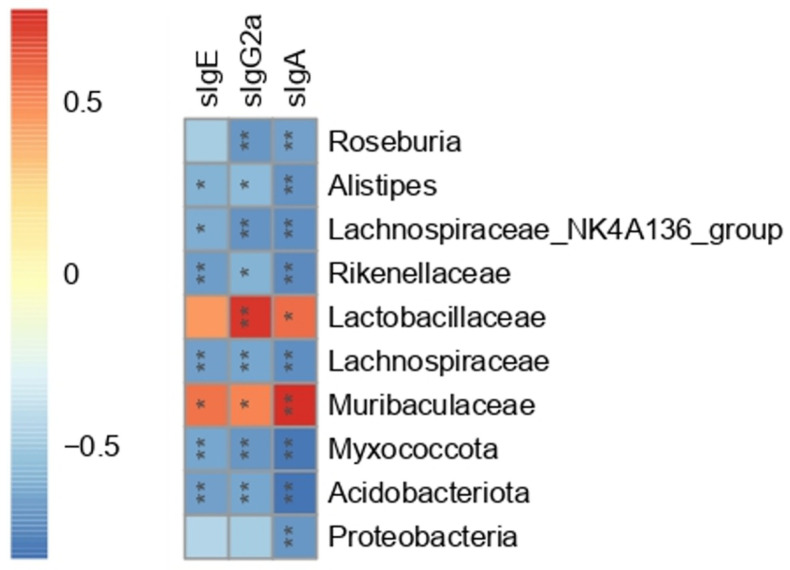
Heat map of Spearman correlation between serum peanut-specific antibodies (sIgE, sIgG2a, and sIgA) and 10 bacterial OTUs at the phylum, family, and genus levels (*n* = 6 for PE group; *n* = 5 for Ad and Ctrl groups). Red represented a positive correlation, and blue represented a negative correlation. ns: no significance; *: *p* < 0.05; **: *p* < 0.01.

To further investigate the association between the gut microbiota and immune system, Spearman correlation analysis was carried out between serum peanut-specific antibodies (sIgE, sIgG2a, and sIgA) and 10 bacterial OTUs. The 10 bacterial OTUs were selected based on Section 3.6. As shown in Figure 7, Acidobacteriota, Myxococcota, Lachnospiraceae, and Rikenellaceae presented a significant negative correlation with sIgE (*p* < 0.01). Muribaculaceae, *Alistipes*, and *Lachnospiraceae_NK4A136_group* were also correlated with sIgE (*p* < 0.05), while Proteobacteria, Lactobacillaceae, and Roseburia showed no correlation with sIgE. Except for Proteobacteria, the rest of the nine bacteria OTUs had significant correlations with sIgG2a (*p* < 0.05). All the 10 bacterial OTUs were significantly correlated with sIgA (*p* < 0.05). These 10 bacteria OTUs showed different correlations with serum antibodies. The Spearman correlation indirectly supported the results of 16S rRNA. Combined with the significant differences in relative abundance between the PE and Ad group, Spearman correlation with serum antibodies, and LDA scores (LDA score threshold > 4), six bacteria OTUs including Acidobacteriota, Lachnospiraceae, Rikenellaceae, Muribaculaceae, *Alistipes*, and *Lachnospiraceae_NK4A136_group* were regarded as the PA-related gut microbial signatures.

### 3.8. PE Sensitization Altered the Predicted Functions of Gut Microbiota

**Figure 8:**
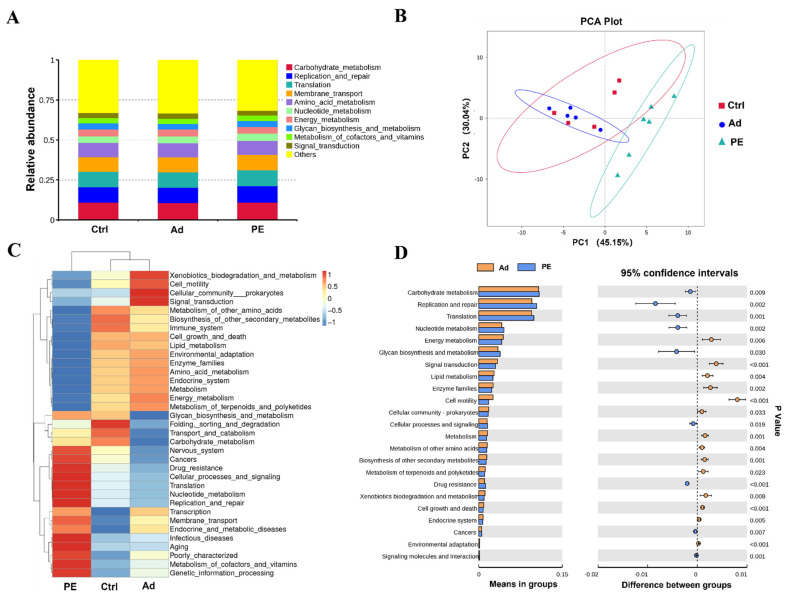
Predicted functions of gut microbiota were analyzed by the Tax4Fun analysis (*n* = 6 for PE group; *n* = 5 for Ad and Ctrl groups). (**A**) Relative abundance of genes in functional pathways on level 2. (**B**) PCA analysis on level 2. (**C**) Heatmap of functional pathways on level 2. (**D**) The significant functional pathways between the PE and Ad groups on level 2. Each dot represented one sample.

To further evaluate the distinction of gut microbiota in functional pathways, the Tax4Fun analysis was performed. Carbohydrate metabolism, replication and repair, translation, membrane transport, and amino acid metabolism were the prominent function pathways of gut microbiota on level 2 (Figure 8A). The cluster of the PE group was separated from that of the Ad and Ctrl groups, which indicated that PE sensitization might result in different functions of gut microbiota (Figure 8B). It was consistent with the results of the heatmap, which presented a marked difference in the relative abundance of genes in function pathways on level 2 (Figure 8C). It was found that the PE group up-regulated the genes of 9 pathways and down-regulated the genes of 14 pathways compared to the Ad group on level 2, among which 9 pathways were associated with metabolism (*p* < 0.05) (Figure 8D). It suggested that metabolism might mediate PA development. Six pathways of metabolism (metabolism of other amino acids, lipid metabolism, enzyme families, energy metabolism, carbohydrate metabolism, and biosynthesis of other secondary metabolites) had lower expression and three pathways (xenobiotics biodegradation and metabolism, nucleotide metabolism, and glycan biosynthesis and metabolism) had higher expression in the PE group compared to the Ad group on level 2 (*p* < 0.05). Moreover, we analyzed the genes in metabolism pathways on level 3. It was found that the relative abundance of genes in purine metabolism (level 3) was significantly increased in the PE group compared to the Ad group (*p* < 0.001) (Figure S2). The pathway of purine metabolism presented a significant positive correlation with sIgE (*p* < 0.05, R = 0.65) (Figure S2). Collectively, these findings indicated that PE sensitization altered the predicted functions of gut microbiota in mice. Since the results of functional predictions were only based on 16S rRNA, further studies are still needed to confirm.

## 4. Discussion

These findings suggested that three sensitizations via intraperitoneal injection with alum and two challenges via oral gavage and intraperitoneal injection could establish a peanut allergy model in BALB/c mice. Additionally, it was found that PE sensitization reduced the α-diversity of gut microbiota and altered the microbiota composition. Six bacterial OTUs at different levels (phylum, family, and genus levels) were closely associated with PA.

The levels of allergen-specific antibodies in serum are commonly used to assess allergen sensitization [33]. PE-sensitized mice showed higher levels of sIgE, sIgG2a, and sIgA in serum. Awatif Lifrani et al. reported increasing levels of sIgE and sIgG in BALB/c mice during peanut sensitization [20]. Cytokines can also reflect the severity of FA. The levels of IL-4 in the duodenum and splenic lymphocytes were increased due to PE sensitization. IL-4 promotes allergic inflammation and induces the development of FA [39]. Moreover, the infiltration of inflammatory cells and mast cells in the duodenum also supported the effectiveness of the peanut allergy model.

Increasing studies have shown that gut microbiota plays a critical role in FA [7]. The data from human studies suggested that gut microbiota dysbiosis may precede the development of FA [40,41]. We found that the administration of PE completely reshaped the gut microbiota in BALB/c mice and significantly reduced the α-diversity of gut microbiota (*p* < 0.05). Infants who suffered from cow’s milk allergy (CMA) presented identical results [42]. Then, we further analyzed the significantly varied bacteria OTUs. PE-sensitized mice had lower expression in Acidobacteriota, Lachnospiraceae Rikenellaceae, *Alistipes*, and *Lachnospiraceae_NK4A136_group* and higher expression in Muribaculaceae compared to the Ad group (*q* < 0.05, LDA > 4). They were all significantly correlated with serum peanut-specific antibodies (*p* < 0.05). The six bacterial OTUs were considered to be the PA-related gut microbial signatures. Muribaculaceae was proved to be associated with ovalbumin (OVA)-sensitized mice and had a negative correlation with OVA-specific IgE and IgG [43], while Muribaculaceae had a positive correlation to serum antibodies in this study (*p* < 0.05). We speculate that this may be due to different allergens. Different IgE-mediated food allergies are significantly different in their gut microbiota [5]. Lachnospiraceae was found to promote the immune tolerance for peanuts, which supported our findings [44]. The level of Rikenellaceae was significantly different between children with FA and the healthy controls [45]. Rikenellaceae, Lachnospiraceae, Lactobacillaceae, and Porphyromonadaceae were also regarded as the specific microbiota signatures in OVA-sensitized Il4raF709 mice [46]. Rikenellaceae was also related to CMA [47]. These studies suggested that Rikenellaceae may play an important role in FA. Additionally, we found that low expression of *Alistipes* and *Lachnospiraceae_NK4A136_group* may contribute to PA. *Alistipes* is a recently isolated bacterial genus from medical clinical samples [48]. It is closely associated with hepatic encephalopathy, cardiovascular disease, and inflammatory bowel disease [48,49,50,51]. Babies with FA lowered the abundance of genera in Bacteroidetes including *Alistipes*, *Parabacteroides*, and *Prevotella* [52]. We reported that *Alistipes* had a significant negative correlation to peanut-specific antibodies (*p* < 0.05). It suggested that *Alistipes* may have the potential to protect against PA. Studies reported that *Lachnospiraceae_NK4A136_group* could produce SCFAs, such as butyric acid [53,54]. SCFAs were proved to alleviate the inflammatory bowel diseases and regulate the intestinal homeostasis in animal models [55]. The level of *Lachnospiraceae_NK4A136_group* was decreased in colitis mice, which was reversed by the intervention of aged ripe Pu-erh tea [53]. The elevated level of *Lachnospiraceae_NK4A136_group* could also ameliorate obesity in mice, likely due to its ability to enhance the gut barrier function [56]. These studies were in line with our findings that the decreased level of *Lachnospiraceae_NK4A136_group* might aggravate PA.

Moreover, the Tax4Fun analysis showed that nine functional pathways related to metabolism (level 2) were significantly altered in PE-sensitized mice (*p* < 0.05). Gut microbiota can function by the metabolites they produce [1]. Egg-allergic people presented low expression of purine metabolism [1]. Similarly, peanut-allergic children and mice showed a significantly increased level of uric acid which was associated with purine metabolism. Uric acid might mediate PE sensitization by activating dendritic cells. The expressions of adenine, adenosine, inosine, hypoxanthine, and xanthine were also significantly altered in peanut-allergic mice [57]. They all played a critical role in purine metabolism. These studies indicated that purine metabolism was likely to mediate the development of FA.

## 5. Conclusions

In this study, PE sensitization caused high levels of serum peanut-specific antibodies (sIgE, sIgG2a, and sIgA), the secretion of Th2-related cytokines (IL-4), and the infiltration of inflammatory cells and mast cells. Moreover, PE sensitization reduced the α-diversity of gut microbiota. Acidobacteriota (phylum level), Muribaculaceae, Lachnospiraceae, Rikenellaceae (family level), and *Alistipes*, *Lachnospiraceae_NK4A136_group* (genus level) may be the characteristics of PA-related gut microbiota in BALB/c mice. Our findings provided new evidence for the identification and classification of characteristic PA-related microbiota, which helped to establish gut microbiota-based therapies for PA.

## Data Availability

The data that support the findings of this study are available from the corresponding author upon reasonable request.

## Supplementary Materials

The following supporting information can be downloaded at: https://www.mdpi.com/article/10.3390/foods11101395/s1, Figure S1: Relative abundance of *Roseburia* and *Rikenella* in mice (*n* = 6 for PE group; *n* = 5 for Ad and Ctrl groups). ns: no significance; *: *q* < 0.05; Figure S2. Predicted functions of gut microbiota were analyzed by the Tax4Fun analysis (*n* = 6 for PE group; *n* = 5 for Ad and Ctrl groups). (A) Relative abundance of genes in purine metabolism pathway on level 3. (B) Spearman correlation between sIgE and relative abundance of genes in purine metabolism pathway. R, the Spearman correlation coefficient. P, the significance of Spearman correlation. ns: no significance; **: *p* < 0.01; ***: *p* < 0.001.

## References

[1] Fazlollahi M., Chun Y., Grishin A., Wood R.A., Burks A.W., Dawson P., Jones S.M., Leung D.Y.M., Sampson H.A., Sicherer S.H. (2018). Early-life gut microbiome and egg allergy. Allergy.

[2] Boyce J.A., Assa’ad A., Burks A.W., Jones S.M., Sampson H.A., Wood R.A., Plaut M., Cooper S.F., Fenton M.J., Arshad S.H. (2010). Guidelines for the Diagnosis and Management of Food Allergy in the United States: Summary of the NIAID-Sponsored Expert Panel Report. J. Allergy Clin. Immunol..

[3] Al-Muhsen S., Clarke A.E., Kagan R.S. (2003). Peanut allergy: An overview. Can. Med. Assoc. J..

[4] Venter C., Sicherer S.H., Greenhawt M. (2019). Management of Peanut Allergy. J. Allergy Clin. Immunol. Pract..

[5] Goldberg M.R., Mor H., Neriya D.M., Magzal F., Muller E., Appel M.Y., Nachshon L., Borenstein E., Tamir S., Louzoun Y. (2020). Microbial signature in IgE-mediated food allergies. Genome Med..

[6] Andreassen M., Rudi K., Angell I.L., Dirven H., Nygaard U.C. (2018). Allergen Immunization Induces Major Changes in Microbiota Composition and Short-Chain Fatty Acid Production in Different Gut Segments in a Mouse Model of Lupine Food Allergy. Int. Arch. Allergy Immunol..

[7] Bunyavanich S., Berin M.C. (2019). Food allergy and the microbiome: Current understandings and future directions. J. Allergy Clin. Immunol..

[8] Sharma G., Im S.H. (2018). Probiotics as a Potential Immunomodulating Pharmabiotics in Allergic Diseases: Current Status and Future Prospects. Allergy Asthma Immunol. Res..

[9] Stefka A.T., Feehley T., Tripathi P., Qiu J., McCoy K., Mazmanian S.K., Tjota M.Y., Seo G.Y., Cao S., Theriault B.R. (2014). Commensal bacteria protect against food allergen sensitization. Proc. Natl. Acad. Sci. USA.

[10] Sivaprakasam S., Prasad P.D., Singh N. (2016). Benefits of short-chain fatty acids and their receptors in inflammation and carcinogenesis. Pharmacol. Ther..

[11] Gao B.B., Wang R.J., Peng Y., Li X.B. (2018). Effects of a homogeneous polysaccharide from Sijunzi decoction on human intestinal microbes and short chain fatty acids in vitro. J. Ethnopharmacol..

[12] Org E., Blum Y., Kasela S., Mehrabian M., Kuusisto J., Kangas A.J., Soininen P., Wang Z.N., Ala-Korpela M., Hazen S.L. (2017). Relationships between gut microbiota, plasma metabolites, and metabolic syndrome traits in the METSIM cohort. Genome Biol..

[13] Walker W.A., Iyengar R.S. (2015). Breast milk, microbiota, and intestinal immune homeostasis. Pediatr. Res..

[14] Cheng J., Ringel-Kulka T., Heikamp-de Jong I., Ringel Y., Carroll I., de Vos W.M., Salojarvi J., Satokari R. (2016). Discordant temporal development of bacterial phyla and the emergence of core in the fecal microbiota of young children. ISME J..

[15] Palmer C., Bik E.M., DiGiulio D.B., Relman D.A., Brown P.O. (2007). Development of the human infant intestinal microbiota. PLoS Biol..

[16] Atarashi K., Tanoue T., Oshima K., Suda W. (2013). Treg induction by a rationally selected mixture of Clostridia strains from the human microbiota. Nature.

[17] Koppelman S.J., Knol E.F., Vlooswijk R.A.A., Wensing M., Knulst A.C., Hefle S.L., Gruppen H., Piersma S. (2003). Peanut allergen Ara h 3: Isolation from peanuts and biochemical characterization. Allergy.

[18] Pons L., Ponnappan U., Hall R.A., Simpson P., Cockrell G., West C.M., Sampson H.A., Helm R.M., Burks A.W. (2004). Soy immunotherapy for peanut-allergic mice: Modulation of the peanut-allergic response. J. Allergy Clin. Immunol..

[19] Leonard S.A., Martos G., Wang W., Nowak-Wegrzyn A., Berin M.C. (2012). Oral immunotherapy induces local protective mechanisms in the gastrointestinal mucosa. J. Allergy Clin. Immunol..

[20] Lifrani A., Dubarry M., Rautureau M., Aattouri N., Boyaka P.N., Tomé D. (2005). Peanut-lupine antibody cross-reactivity is not associated to cross-allergenicity in peanut-sensitized mouse strains. Int. Immunopharmacol..

[21] Liu Y., Zheng S.J., Cui J.L., Guo T.T., Zhang J.T., Li B.L. (2021). Alleviative Effects of Exopolysaccharide Produced by Lactobacillus helveticus KLDS1.8701 on Dextran Sulfate Sodium-Induced Colitis in Mice. Microorganisms.

[22] Edgar R.C. (2013). UPARSE: Highly accurate OTU sequences from microbial amplicon reads. Nat. Methods.

[23] Li B., Zhang X.X., Guo F., Wu W.M., Zhang T. (2013). Characterization of tetracycline resistant bacterial community in saline activated sludge using batch stress incubation with high-throughput sequencing analysis. Water Res..

[24] Lozupone C.A., Hamady M., Kelley S.T., Knight R. (2007). Quantitative and qualitative beta diversity measures lead to different insights into factors that structure microbial communities. Appl. Environ. Microbiol..

[25] White J.R., Nagarajan N., Pop M. (2009). Statistical methods for detecting differentially abundant features in clinical metagenomic samples. PLoS Comput. Biol..

[26] Palladino C., Breiteneder H. (2018). Peanut allergens. Mol. Immunol..

[27] Md A., Maeda M., Matsui T., Takasato Y., Ito K., Kimura Y. (2021). Purification and molecular characterization of a truncated-type Ara h 1, a major peanut allergen: Oligomer structure, antigenicity, and glycoform. Glycoconj. J..

[28] Rabjohn P., Helm E.M., Stanley J.S., West C.M., Sampson H.A., Burks A.W., Bannon G.A. (1999). Molecular cloning and epitope analysis of the peanut allergen Ara h 3. J. Clin. Investig..

[29] Ratnaparkhe M.B., Lee T.H., Tan X., Wang X.Y., Li J.P., Kim C., Rainville L.K., Lemke C., Compton R.O., Robertson J. (2014). Comparative and Evolutionary Analysis of Major Peanut Allergen Gene Families. Genome Biol. Evol..

[30] Rao H., Tian Y., Fu W.H., Xue W.T. (2018). In vitro digestibility and immunoreactivity of thermally processed peanut. Food Agric. Immunol..

[31] Kleber-Janke T., Crameri R., Appenzeller U., Schlaak M., Becker W.M. (1999). Selective cloning of peanut allergens, including profilin and 2S albumins, by phage display technology. Int. Arch. Allergy Immunol..

[32] Petersen A., Kull S., Rennert S., Becker W.M., Krause S., Ernst M., Gutsmann T., Bauer J., Lindner B., Jappe U. (2015). Peanut defensins: Novel allergens isolated from lipophilic peanut extract. J. Allergy Clin. Immunol..

[33] Castan L., Bogh K.L., Maryniak N.Z., Epstein M.M., Kazemi S., O’Mahony L., Bodinier M., Smit J.J., van Bilsen J.H.M., Blanchard C. (2020). Overview of in vivo and ex vivo endpoints in murine food allergy models: Suitable for evaluation of the sensitizing capacity of novel proteins?. Allergy.

[34] Gould H.J., Sutton B.J., Beavil A.J., Beavil R.L., McCloskey N., Coker H.A., Fear D., Smurthwaite L. (2003). The biology of IgE and the basis of allergic disease. Annu. Rev. Immunol..

[35] Shamji M.H., Valenta R., Jardetzky T., Verhasselt V., Durham S.R., Wurtzen P.A., van Neerven R.J.J. (2021). The role of allergen-specific IgE, IgG and IgA in allergic disease. Allergy.

[36] Pali-Scholl I., Szollosi H., Starkl P., Scheicher B., Stremnitzer C., Hofmeister A., Roth-Walter F., Lukschal A., Diesner S.C., Zimmer A. (2015). Protamine nanoparticles with CpG-oligodeoxynucleotide prevent an allergen-induced Th2-response in BALB/c mice. Eur. J. Pharm. Biopharm..

[37] Swain S.L., Weinberg A.D., English M., Huston G. (1990). Il-4 Directs the Development of Th2-like Helper Effectors. J. Immunol..

[38] Wang K., Yang Q.Q., Ma Q.X., Wang B., Wan Z.R., Chen M.L., Wu L.M. (2018). Protective Effects of Salvianolic Acid A against Dextran Sodium Sulfate-Induced Acute Colitis in Rats. Nutrients.

[39] Iwaszko M., Bialy S., Bogunia-Kubik K. (2021). Significance of Interleukin (IL)-4 and IL-13 in Inflammatory Arthritis. Cells.

[40] Nakayama J., Kobayashi T., Tanaka S., Korenori Y., Tateyama A., Sakamoto N., Kiyohara C., Shirakawa T., Sonomoto K. (2011). Aberrant structures of fecal bacterial community in allergic infants profiled by 16S rRNA gene pyrosequencing. FEMS Immunol. Med. Microbiol..

[41] Di Costanzo M., Carucci L., Canani R.B., Biasucci G. (2020). Gut Microbiome Modulation for Preventing and Treating Pediatric Food Allergies. Int. J. Mol. Sci..

[42] Jing W., Liu Q.B., Wang W. (2021). Bifidobacterium bifidum TMC3115 ameliorates milk protein allergy in by affecting gut microbiota: A randomized double-blind control trial. J. Food Biochem..

[43] Xu J., Ye Y., Ji J., Sun J., Wang J.S., Sun X. (2022). Untargeted Metabolomic Profiling Reveals Changes in Gut Microbiota and Mechanisms of Its Regulation of Allergy in OVA-Sensitive BALB/c Mice. J. Agric. Food Chem..

[44] He Z.Y., Vadali V.L.G., Szabady R.L., Zhang W.M., Norman J.M., Roberts B., Tibshirani R., Desai M., Chinthrajah R.S., Galli S.J. (2021). Increased diversity of gut microbiota during active oral immunotherapy in peanut-allergic adults. Allergy.

[45] Kourosh A., Luna R.A., Balderas M., Nance C., Anagnostou A., Devaraj S., Davis C.M. (2018). Fecal microbiome signatures are different in food-allergic children compared to siblings and healthy children. Pediatr. Allergy Immunol..

[46] Rivas M.N., Burton O.T., Wise P., Zhang Y.Q., Hobson S.A., Lloret M.G., Chehoud C., Kuczynski J., DeSantis T., Warrington J. (2013). A microbiota signature associated with experimental food allergy promotes allergic sensitization and anaphylaxis. J. Allergy Clin. Immunol..

[47] Vonk M.M., Engen P.A., Naqib A., Green S.J., Keshavarzian A., Blokhuis B.R.J., Garssen J., Knippels L.M.J., van Esch B.C.A.M. (2020). Altered microbial community structure and metabolism in cow’s milk allergic mice treated with oral immunotherapy and fructo-oligosaccharides. Benef. Microbes.

[48] Parker B.J., Wearsch P.A., Veloo A.C.M., Rodriguez-Palacios A. (2020). The Genus Alistipes: Gut Bacteria with Emerging Implications to Inflammation, Cancer, and Mental Health. Front. Immunol..

[49] Zuo K., Li J., Li K.B., Hu C.W., Gao Y.F., Chen M.L., Hu R.M., Liu Y., Chi H.J., Wang H.J. (2019). Disordered gut microbiota and alterations in metabolic patterns are associated with atrial fibrillation. Gigascience.

[50] Frank D.N., Amand A.L.S., Feldman R.A., Boedeker E.C., Harpaz N., Pace N.R. (2007). Molecular-phylogenetic characterization of microbial community imbalances in human inflammatory bowel diseases. Proc. Natl. Acad. Sci. USA.

[51] Ran B.B., Guo C.E., Li W.D., Li W.S., Wang Q., Qian J.X., Li H.L. (2021). Sea buckthorn (*Hippophae rhamnoides* L.) fermentation liquid protects against alcoholic liver disease linked to regulation of liver metabolome and the abundance of gut microbiota. J. Sci. Food Agric..

[52] Rachid R.A., Gerber G., Li N., Umetsu D.T., Bry L., Chatila T.A. (2016). Food Allergy in Infancy Is Associated with Dysbiosis of the Intestinal Microbiota. J. Allergy Clin. Immunol..

[53] Hu S.S., Li S., Liu Y., Sun K., Luo L.Y., Zeng L. (2021). Aged Ripe Pu-erh Tea Reduced Oxidative Stress-Mediated Inflammation in Dextran Sulfate Sodium-Induced Colitis Mice by Regulating Intestinal Microbes. J. Agric. Food Chem..

[54] Hu S.W., Yang H.C., Gao X., Li S.J., Jiang W., Liu Y. (2020). Egg oil from *Portunus trituberculatus* alleviated obesity and regulated gut microbiota in mice. Sci. Rep..

[55] Sun M.M., Wu W., Liu Z.J., Cong Y.Z. (2017). Microbiota metabolite short chain fatty acids, GPCR, and inflammatory bowel diseases. J. Gastroenterol..

[56] Ma L.Y., Ni Y.H., Wang Z., Tu W.Q., Ni L.Y., Zhuge F., Zheng A.Q., Hu L.T., Zhao Y.F., Zheng L.J. (2020). Spermidine improves gut barrier integrity and gut microbiota function in diet-induced obese mice. Gut Microbes.

[57] Kong J., Chalcraft K., Mandur T.S., Jimenez-Saiz R., Walker T.D., Goncharova S., Gordon M.E., Naji L., Flader K., Larche M. (2015). Comprehensive metabolomics identifies the alarmin uric acid as a critical signal for the induction of peanut allergy. Allergy.

