# Supplementary material for: Gut Microbial Signatures Associated with Peanut Allergy in a BALB/c Mouse Model
Source: Foods. 2022 May 12;11(10):1395. doi: 10.3390/foods11101395 (PMC9141413; doi:10.3390/foods11101395)
Supplement: Supplementary file 1 [file foods-11-01395-s001.zip › foods-1695028-supplementary.pdf]

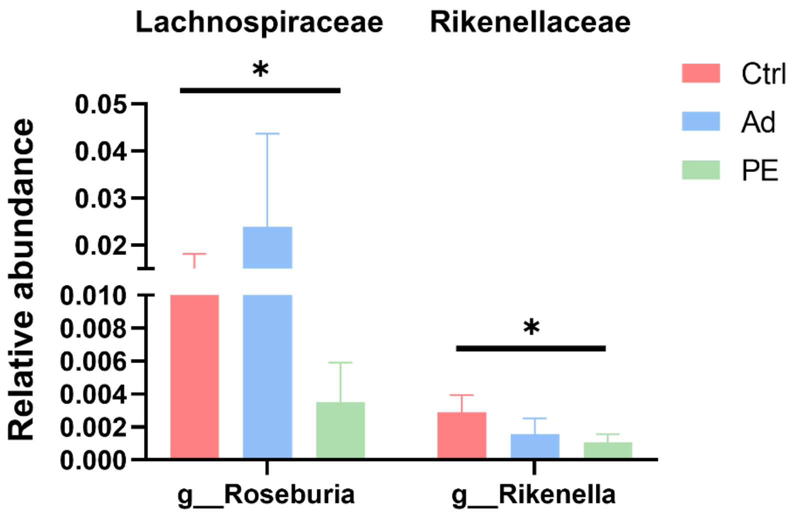

Figure S1. Relative abundance of *Roseburia* and *Rikenella* in mice (n = 6 for PE group; n = 5 for Ad and Ctrl groups). ns: no significance; *: $q < 0.05$.

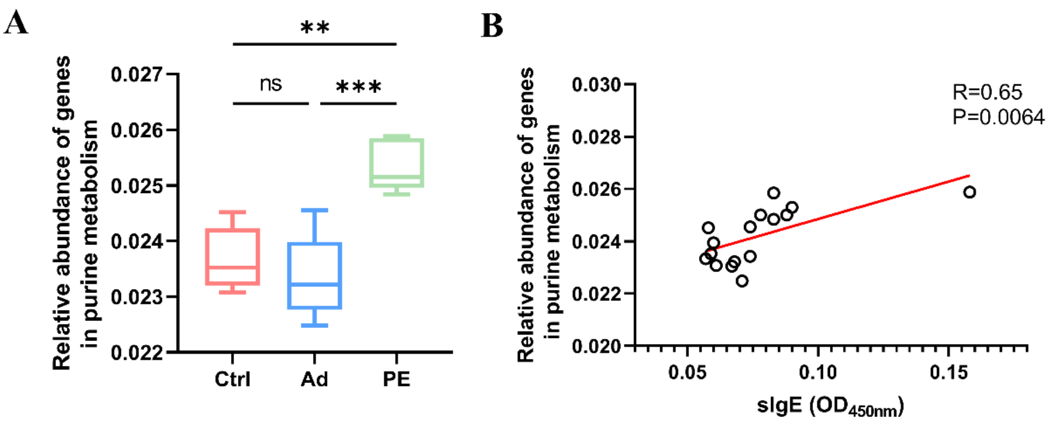

Figure S2. Predicted functions of gut microbiota were analyzed by the Tax4Fun analysis (n = 6 for PE group; n = 5 for Ad and Ctrl groups). (A) Relative abundance of genes in purine metabolism pathway on level 3. (B) Spearman correlation between sIgE and relative abundance of genes in purine metabolism pathway. R, the Spearman correlation coefficient. P, the significance of Spearman correlation. ns: no significance; **: $p < 0.01$; ***: $p < 0.001$.
